# The acupoint herbal plaster for the prevention and treatment of postoperative nausea and vomiting after PLIF with general anesthesia: study protocol for a multicenter randomized controlled trial

**DOI:** 10.1186/s13063-021-05037-7

**Published:** 2021-01-22

**Authors:** Huiqing Xu, Xu Wei, Ranxing Zhang, Ling Li, Zhijun Zhang, Ruo Jia, Xiaofei Zhang, Xiumei Gao, Xicheng Dong, Junjun Pan

**Affiliations:** 1 Department of Anesthesiology, Wangjing Hospital, China Academy of Chinese Medical Sciences, Beijing, 100102 China; 2 Department of Scientific Research, Wangjing Hospital, China Academy of Chinese Medical Sciences, Beijing, 100102 China; 3 Department of Clinical Laboratory, Wangjing Hospital, China Academy of Chinese Medical Sciences, Beijing, 100102 China; 4 Department of Anesthesiology, Xiyuan Hospital, China Academy of Chinese Medical Sciences, Beijing, 100091 China; 5 Department of Anesthesiology, Guang’anmen Hospital, China Academy of Chinese Medical Sciences, Beijing, 100053 China; 6 TCM Characteristic Clinical Center, Wangjing Hospital, China Academy of Chinese Medical Sciences, 100102 Beijing, China

**Keywords:** Chinese medicine, Acupoint herbal plaster, Postoperative nausea and vomiting, Randomized controlled trial

## Abstract

**Background:**

Postoperative nausea and vomiting (PONV) are common in posterior lumbar intervertebral fusion (PLIF) patients undergoing general anesthesia. The previous clinical observation has shown that a traditional acupoint herbal plaster (AHP) is beneficial to patients with PONV. This trial aims to assess the effect of the AHP for the prevention and treatment of PONV after PLIF in patients with general anesthesia.

**Methods:**

A multicenter, parallel, randomized controlled trial (RCT) will be conducted. A total of 166 participants will be randomized to either a treatment group receiving an AHP or a control groups receiving an acupoint placebo plaster (APP) in a 1:1 ratio. The primary outcomes are the first occurrence and frequency of nausea and vomiting. The secondary outcomes include the severity grading of nausea and vomiting using a visual analog scale (VAS) measurement system, quality of life, and serological indicators. The safety evaluation is mainly about adverse events and skin reactions’ observation. Assessments will be carried out at the baseline, day 1, and day 2 (the end of the intervention). The central randomization system in the clinical trial (http://124.205.181.142:8082/xwtf/) will be used to conduct random allocation.

**Discussion:**

This scientific methodology design of the trial is expected to provide clinical evidence to support the AHP for the prevention and treatment of PONV.

**Trial registration:**

This study is retrospectively registered with the Chinese Clinical Trial Registry (http://www.chictr.org.cn) on 19 April 2018. ID: ChiCTR1800015768.

## Administrative information

The order of the items has been modified to group similar items (see http://www.equator-network.org/reporting-guidelines/spirit-2013-statement-defining-standard-protocol-items-for-clinical-trials/).

**Table:** 

Title {1}	Acupoint herbal plaster for the prevention and treatment of postoperative nausea and vomiting after PLIF with general anesthesia: study protocol for a multicenter randomized controlled trial
Trial registration {2a and 2b}.	{2a,2b} This study is registered with the Chinese Clinical Trial Registry (http://www.chictr.org.cn) on 19 April 2018. ID: ChiCTR1800015768.
Protocol version {3}	Version 2.0,03/14/2018.
Funding {4}	This study is supported by a joint project from the China Academy of Chinese Medical Sciences (CACMS) (ZZ11-033). According to the general plan of basic scientific research operating expenses and annual key work arrangement of CACMS, the joint innovation projects and other projects of CACMS have been approved after expert demonstration and evaluation.
Author details {5a}	Huiqing Xu1†, Xu Wei2†, Ranxing Zhang3, Ling Li1, Zhijun Zhang1, Ruo jia1, Xiaofei Zhang1, Xiumei Gao4, Xicheng Dong5, Junjun pan6* 1 Department of Anesthesiology, Wangjing Hospital, China Academy of Chinese Medical Sciences, Beijing 100102, China. 2 Department of Scientific Research, Wangjing Hospital, China Academy of Chinese Medical Sciences, Beijing 100102, China. 3 Department of Clinical Laboratory, Wangjing Hospital, China Academy of Chinese Medical Sciences, Beijing 100102, China. 4 Department of Anesthesiology, Xiyuan Hospital, China Academy of Chinese Medical Sciences, Beijing 100091, China. 5 Department of Anesthesiology, Guang’anmen Hospital, China Academy of Chinese Medical Sciences, Beijing 100053, China. 6 Characteristic treatment center, Wangjing Hospital, China Academy of Chinese Medical Sciences, Beijing 100102, China.
Name and contact information for the trial sponsor {5b}	Huiqing Xu, e-mail: dr_xuhuiqing@sina.com.
Role of sponsor {5c}	Huiqing Xu is the Chief Director of Anesthesiology Department, Wangjing Hospital, China Academy of Chinese Medical Sciences, Beijing 100102, China. Huiqing Xu will supervise and coordinate the clinical trial. Huiqing Xu and Xu Wei contributed equally to this work.

## Introduction

### Background and rationale {6a}

Postoperative nausea and vomiting (PONV) are some of the most common and undesirable side effects of conventional anesthesia surgery [1–3]. The incidence of PONV after general anesthesia may reach as high as 30~50% [4]. PONV could also lead to serious complications, such as hematoma, surgical incision hemorrhage, pulmonary aspiration, wound dehiscence, and dehydration, aspiration pneumonia, and increased intracranial pressure [5–8]. On the other hand, PONV was likely to reduce patients’ satisfaction, delay discharge and recovery, and increase medical costs [9, 10]. Therefore, unignorable complication still poses a severe challenge for anesthesiologists, clinicians, and researchers.

Existing consensus guidelines from the American Society for enhanced recovery (ASER) recommended a variety of medications that worked at different receptor sites, such as antihistamines, steroids, and so on [11]. However, no single antiemetic drug could fully respond to prophylaxis and management of PONV, and high-risk individuals often did not receive appropriate antiemetic prophylaxis [12]. Besides, traditional drug therapy had its obvious side effects, while the costs of new agents including NK1-antagonists or the newer 5-HT3 antagonists became relatively higher [13, 14]. On the contrary, more and more turned to alternative and complementary medicine, including herbal medications, acupressure, and so on [15–17].

Non-pharmacologic therapies are also a considerable therapeutic method for PONV [11]. Among the therapeutic methods, traditional Chinese medicine (TCM) therapy regimen including acupuncture, acupressure, and acupoint herbal plaster (AHP) was commonly used based on the treatment principle of “descending stomach qi”. Because the postoperative patients could not immediately eat more within 6 to 8 h, so oral Chinese medicine is not often taken in the treatment of PONV. AHP, by contrast, became a relatively appropriate choice and was easier for patients to accept. As one type of TCM method, herbal plaster was applied with a drug applicator using the mature technique, stimulating the skin at specific acupoints [18]. Our team previously completed the 36 cases clinical observation of AHP for the prevention and treatment of PONV after surgery [19]. It was found that AHP at Neiguan (PC6) and Zhongwan (CV12) had a definite effect in relieving the symptoms of PONV. The Chinese herbal formula which is used in the plaster consist of three commonly used herbs: ginger pinellia (Jiangbanxia, Pinelliae Rhizoma Praeparatumcum Zingibere Et Alumine), ginger (Shengjiang, Zingiberis Rhizoma Recens), and *Syzygium aromaticum* (Dingxiang, Caryophylli Flos). And the formula contained *Xiao Banxia decoction* (ginger pinellia and ginger) which was documented in *Shang Han Za Bing Lun*, a classic medical book dating as far back as the Han Dynasty [20]. It could be considering treating all kinds of vomiting. Additionally, PC6 and CV12 had been also the primary choice for the treatment of nausea and vomiting and closely related to gastrointestinal function [21–23].

Having given this, we make the further design and conduct the randomized controlled trial (RCT) to assess the efficacy and safety of AHP for PONV. As far as we know, this study is the first RCT to verify the effect of AHP for the prevention and treatment of PONV.

### Objectives {7}

The hypotheses are as follows.

Confirmatory test for AHP versus APP:

- H0: Effect of AHP ≤ Effect of APP
- H1: Effect of AHP > Effect of APP

### Trial design {8}

**Fig. 1:**
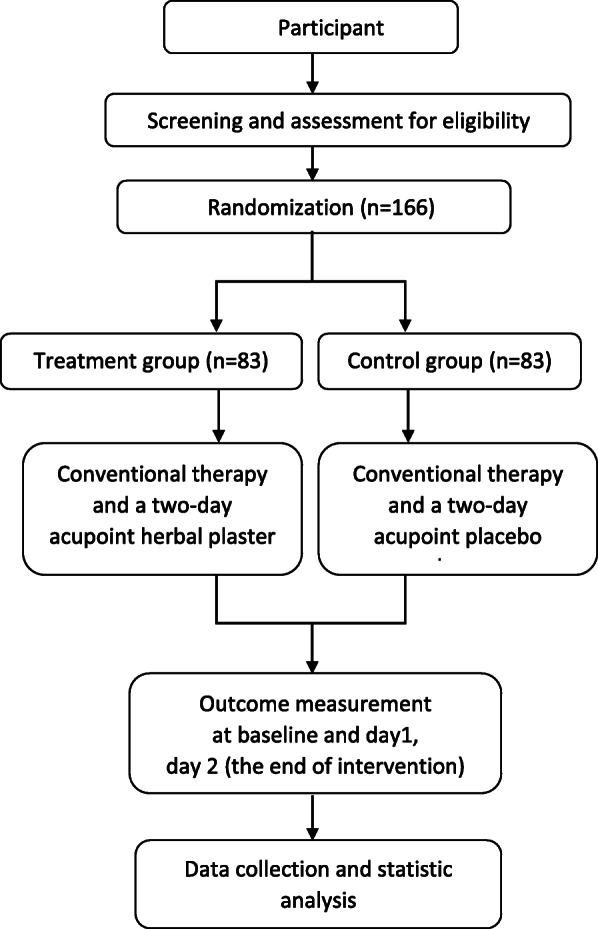
Flow diagram of enrollment, interventions, and assessments

This study is a multicenter, prospective, and double-blind RCT. Ethical approval has been obtained from the ethical committee of Wangjing Hospital, China Academy of Chinese Medical Sciences (No. WJEC-KT-2018-001-P002). This study is registered on chictr.org.cn (ChiCTR1800015768). A brief flow diagram of the entire study is shown in Fig. 1.

**Fig. 2:**
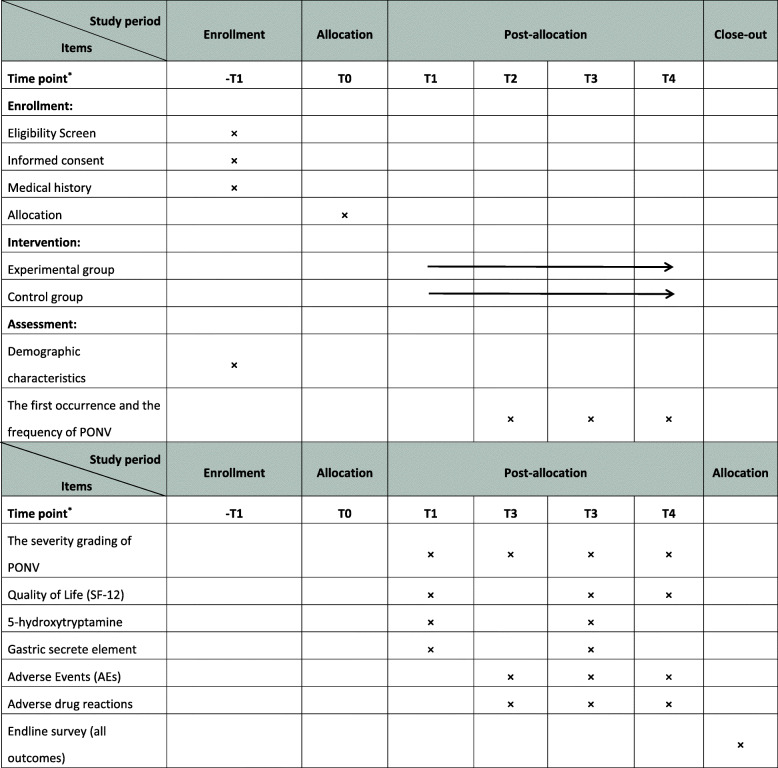
SPIRIT Checklist. T1, pre-operation; T2, postoperative 8 h; T3, postoperative 24 h; T4, postoperative 48 h

The screening and enrollment will be done before the day of treatment on day zero. All the treatments will be scheduled from day 0 to day 2. The study protocol procedure includes the recommended elements in the Standard Protocol Items: Recommendations for Interventional Trials (SPIRIT) checklist [24]. The schedule of events is provided in Fig. 2.

## Methods: participants, interventions, and outcomes

### Study setting {9}

The patients will be recruited from the orthopedic inpatient department from the three affiliated hospitals of China Academy of Chinese Medical Sciences, including Wangjing Hospital, Xiyuan Hospital, and Guanganmen Hospital.

### Eligibility criteria {10}

The participants who meet the following criteria are eligible: (1) PLIF with general anesthesia, (2) aged from 30 to 65 years old, (3) body mass index ranged from 20 to 25 kg/m^2^, and (4) anesthesia grade scale of the American society of anesthesiologists (ASA) judged as I or II Level.

### Who will take informed consent? {26a}

Informed consent will be obtained from all participants before entry into the trial including the possible risks, benefits, and knowledge of the purpose of the trial.

### Additional consent provisions for collection and use of participant data and biological specimens {26b}

This study involves a questionnaire survey and blood sample collection, which is carried out at the same time by medical examination before and 24 h after surgery. Five milliliters of serum will be collected twice, and the remaining blood samples will be treated as abandoned blood after the test. The blood samples of the subjects will be indicated by the study number instead of the name. Unless the consent of the subjects is obtained, the information that can identify the subjects will not be disclosed to members outside the study group.

All members of the study and the sponsor are required to keep the identity of the subjects confidential, and the files will be kept in a locked file cabinet for the researchers’ reference only. To ensure that the research is carried out under the regulations, if necessary, the management department of the research institute or members of the ethics committee may consult the personal data of the subjects in the research unit. When the results of the study were published, no information about the subjects was disclosed.

### Interventions

#### An explanation for the choice of comparators {6b}

All the participants who provide informed consent will be randomized into either a treatment group receiving a 2-day AHP or a control group receiving a 2-day APP. A block randomization sequence using a web-based allocation system will be generated by a central randomization system. The system is managed by the Institute of Clinical Basic Medicine, China Academy of Chinese Medical Sciences. Three practitioners from the different units will apply for the random number from the randomization system, and treatment allocation is determined based on the web the day before the operation.

All the plasters used in the RCT have a similar appearance, smell, and size to ensure the practitioners and patients impossible to distinguish the treatment allocation. Furthermore, the plasters will be packed and labeled to ensure that the researchers and the subjects involved in the study will remain fully blinded as to the identity of the treatment administered. Outcome assessors, laboratory technicians, data managers, and statisticians will not know the treatment allocations, which will be revealed at the end of the trial.

#### Intervention description {11a}

According to the domestic and international expert opinion, all the enrolled patients are given 4-mg Torasetron hydrochloride intravenously at the end of operation [4, 11]. All treatment details will be standardized between practitioners by guiding videos and relevant training before the first treatment session. The acupoint location is based on Term and Location of Acupoints (GB/T12346-2006) issued by the State administration of standardization. The plasters will be attached on the acupoints of bilateral PC6 and RN12 for at most 6 h each time.

##### Experimental group

The patients in the experimental group will receive a 2-day AHP plus conventional Torasetron hydrochloride therapy. Treatment procedures in sequential order are to locate the acupoints, disinfect the skin, and attach the plaster on the skin over the acupoints. It must be removed immediately if an allergic reaction occurs, which may be described by patients as a burning sensation, severe itchiness, blisters, pruritus, or rash on the local skin. A total of 2 sessions will be performed 24 h and 48 h after the operation. The treatment starts before anesthesia before the operation, with at most 6 h of treatment twice per day.

##### Control group

The patients in the control group will receive a 2-day APP plus conventional Torasetron hydrochloride therapy. The treatment procedures between the groups are identical; the only variable is the ingredients of paste.

### Preparation of the plasters

**Fig. 3:**
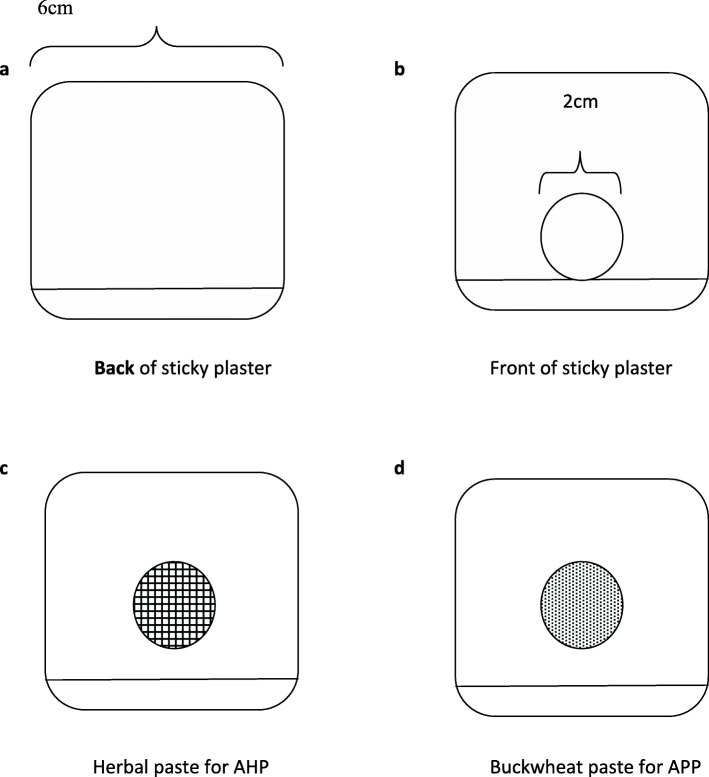
Illustration of the sticky plaster. **a**, **b** The anterior and posterior appearance of the acupoint plaster, respectively. **c**, **d** The paste inside the plaster. **c** The herbal plaster. **d** The buckwheat plaster. These two kinds of paste are identical in appearance and smell. Figures are only for illustrative purposes

In this study, there are two types of paste to be applied on the sticky plasters: herbal paste for the AHP group, and buckwheat paste for the APP group (Fig. 3c and d). In the previous clinical observations, we adapted this formula and standardized its performance. AHP and APP in the trial will be manufactured by the School of Chinese Materia Medica of Beijing University of Chinese Medicine based on the good manufacturing practice (GMP) standards.

In the AHP group, the standard operating procedures are listed as follows.

1. Prepare the herbal materials: the formula includes ginger pinellia, ginger, and *Syzygium aromaticum*. The ingredients of this formula are mixed in a weight ratio of 1:1:1 and then the raw materials ground into a fine powder (200 mesh screen) using a special Chinese medicinal crop powder mixer.
2. Add the liquid base: a total of 100-g mixed herbal powder is put into a container. Fifty-gram honey is added and stirred well to make a thick herbal paste.
3. Make the sticky plaster: a round powder cake of the herbal paste is placed on the medical desensitization sticky plaster. The sticky plasters are sized 6 × 6 cm^2^ with a diameter of 2 cm and a thickness of 0.2 cm in the middle of which is placed the host herbal paste (Fig. 3a and b). The rest of the sticky plaster carries no herbal paste, which makes it adhere to the skin over the acupoints.

For the APP group, the placebo paste is made of buckwheat flour with 5% herbal medicines original concentration. It is still mixed with honey to maintain a similar appearance and smell with the AHP. So, the plasters used in two groups are undistinguished to the patients and practitioners.

### Criteria for discontinuing or modifying allocated interventions {11b}

1. The trial can be stopped in case of other diseases or aggravation during the trial.
2. The trial can be stopped in case of emergency or serious adverse events.
3. If the patient withdraws from the trial or fails to continue the trial, or fails to cooperate with the treatment, the trial can be stopped.
4. During the period of patients participating in the trial, the occurrence of infection affects the data of this trial, and the trial can be stopped.

### Strategies to improve adherence to interventions {11c}

The whole research team including the investigators, research assistants, and research nurses will be required to attend the professional training before recruitment. It is beneficial to ensure the researchers are familiar with the trial administration process and strictly adhere to the study protocol. All the blood samples will be analyzed and check in the research laboratory of Beijing north biotechnology every week. The data collected in this trial will comprise information recorded in the case report forms. Data quality will be checked regularly by research assistants and overseen by monitors. Data monitoring will be conducted regularly with standard operating procedures. All the data modifications are to be marked in the case report forms. If participants withdraw from the trial during the study period, the reasons must be documented and the dropout rate is counted.

### Relevant concomitant care permitted or prohibited during the trial {11d}

All the patients will be operated on by the same general anesthesia. The operation is lumbar surgery. The postoperative medication and nursing will be the same.

### Provisions for post-trial care {30}

No obvious toxic and side effects have been found in the existing studies, but in the course of the study, abnormal damage or unexpected adverse reactions or serious adverse reactions are not excluded, and individual groups are not tolerant or allergic to individual drugs or patches. Adverse reactions related to this study may be observed at any time. In case of any discomfort, new change of condition, or any unexpected situation, whether related to treatment or not, the participant shall inform the investigator in time, and he will make the judgment and medical treatment. Investigators will do their best to prevent and treat. Possible harm due to this study. If there are adverse events related to this study in the clinical trial, the medical expert committee will determine whether it is related to the trial scheme according to law.

### Outcomes {12}

#### Primary outcome

##### The first occurrence and frequency of PONV

The primary outcome will be recorded within the postoperative 48 h. The important index will be self-reported daily and recorded in a diary during the treatment course. In the meantime, each participant will be face-to-face interviewed to attend an in-person assessment at the orthopedic inpatient department.

#### Secondary outcomes

During different time points, the participants will be asked to complete specified physiological tests and self-reported questionnaires.

##### The severity grading of PONV

The severity will be self-assessed using the scores from the visual analog scale (VAS) at pre-operation, postoperative 8 h, 24 h, and 48 h. VAS allows patients to rate the intensity of their symptoms (scored 0, none; scored 1~3, mild; scored 4~6, moderate; scored 7~10, severe).

##### Quality of life

The quality of life will be self-administered using the functional assessment of the SF-12 questionnaire at pre-operation, postoperative 24 h and 48 h. The SF-12 questionnaire measures 12 items, which can be classified as a physical and mental component [25].

##### Blood tests

Blood samples will be collected at pre-operation and postoperative 24 h to measure the levels of serological indicators. The fasting venous blood will be drawn and centrifuged, the separated serum is stored at a – 80 °C refrigerator. Gastric secrete elements and 5-hydroxytryptamine will be analyzed in the research laboratory of Beijing North Biotechnology every week.

### Safety assessment

Adverse events (AEs) are defined as any undesirable experience participants endure during the trial period, regardless of whether or not it is associated with the intervention. The participants will be instructed to report AEs to the research team, and a research nurse will monitor participants for potential occurrences of AEs. All details of AEs, such as time of occurrence, severity, management, and causality to the intervention, will be recorded in the case report forms. All AEs will be followed up from the beginning until resolution.

Current evidence has shown that AHP has a lower rate of adverse drug reactions (ADRs) [19]. However, our trial still pays more attention to possible ADRs, particularly associated with skin lesions (urticaria and pruritus) or other symptoms during the study period.

### Participant timeline {13}

The schedule of events is provided in Fig. 2.

### Sample size {14}

A relatively small number of clinical trials has investigated the effects of acupoint plaster on PONV; however, the research results are not appropriate to calculate the sample size for this study. The sample size is calculated based on the primary endpoint. The small sample-size clinical practice suggests the decreased rate of nausea and vomiting frequency is 60% in the treatment group, while the rate is 40% in the control group based on previous literature. According to the superiority test, it is estimated that at least 69 participants per group are needed when the study achieves 90% power and a two-sided 5% significance level. Given the dropout rate to 20%, the sample size in the clinical trial sets at a total of 166 cases, and each group is 83 cases.

### Recruitment {15}

Patients who are about to have PLIF with general anesthesia and meet the inclusion criteria will be recruited through multimodal strategies, including advertisements in hospital social media (WeChat) and newspapers, publicity at community service centers, and a clinical patient database. Informed consent will be obtained before randomization. The schedule of enrolment, intervention, and assessments is shown in Fig. 2.

### Assignment of interventions: allocation

#### Sequence generation {16a}

Eligible patients will be randomly assigned to the treatment group or control group in a 1:1 ratio using a central web-based randomization tool. A block randomization sequence using a web-based allocation system will be generated by a central randomization system (http://124.205.181.142:8082/xwtf/).

#### Concealment mechanism {16b}

The system is managed by an independent statistician (the Institute of Clinical Basic Medicine, China Academy of Chinese Medical Sciences, China), who is not involved in the implementation of statistical analysis of the trial.

#### Implementation {16c}

Three practitioners from the different units will apply for the random number from the randomization system and treatment allocation is determined based on the web the day before the operation.

#### Assignment of interventions: blinding

##### Who will be blinded {17a}

All the plasters used in the RCT have a similar appearance, smell, and size to ensure the practitioners and patients impossible to distinguish the treatment allocation. Furthermore, the plasters will be packed and labeled to ensure that the researchers and the subjects involved in the study will remain fully blinded as to the identity of the treatment administered. Outcome assessors, laboratory technicians, data managers, and statisticians will not know the treatment allocations, which will be revealed at the end of the trial.

##### Procedure for unblinding if needed {17b}

In case of an emergency, the researcher shall open it according to the procedures specified in the test plan. Once the case number is opened, the trial will be suspended, and the researcher shall record the reason for suspension in the case report form.

#### Data collection and management

##### Plans for assessment and collection of outcomes {18a}

The whole research team including the investigators, research assistants, and research nurses will be required to attend the professional training before recruitment. It is beneficial to ensure the researchers are familiar with the trial administration process and strictly adhere to the study protocol.

### Plans to promote participant retention and complete follow-up {18b}

The data collected in this trial will comprise information recorded in the case report forms. Data quality will be checked regularly by research assistants and overseen by monitors. Data monitoring will be conducted regularly with standard operating procedures. All the data modifications are to be marked in the case report forms. If participants withdraw from the trial during the study period, the reasons must be documented and the dropout rate is counted.

### Data management {19}

Data management was handled by the Institute of clinical basic medicine, China Academy of Chinese Medical Sciences. All the trial data are collected through strict verification and management; the database will be locked after all data have been cleaned. And the data are put into an Epidata 3.1 database by two researchers independently. A consistency test is conducted between the database.

### Confidentiality {27}

Both paper files and electronic data will be preserved for at least 5 years after publication. If readers and reviewers have any questions, they can contact the corresponding author for access to the original data. Patient information will remain anonymous, including name, ID number, and telephone number. The management department of the research institute or members of the ethics committee will review the progress of the trial after 3 months, independently of the researchers, and decide if premature closure of the study is required, based solely on adverse events.

### Plans for collection, laboratory evaluation, and storage of biological specimens for genetic or molecular analysis in this trial/future use {33}

Not applicable. The remaining blood samples will be treated as abandoned blood after the test.

### Statistical methods

#### Statistical methods for primary and secondary outcomes {20a}

The efficacy and safety analysis will be conducted according to the intention-to-treat principle. As the third party, the statistical analysis will be performed by the Institute of Clinical Basic Medicine, China Academy of Chinese Medical Sciences, using the statistical packages of social sciences software (SPSS; version 19.0). All the results are based on two-sided tests. Continuous data will be presented as means and standard deviations and compared using the independent *t* test or Wilcoxon’s rank-sum test. On the other hand, categorical data will be presented as percentages and frequencies and analyzed using the chi-square or Fisher’s exact test. The missing values will be implemented by the method of multiple imputations. A *p* value less than or equal to 0.05 will be considered statistically significant.

#### Interim analyses {21b}

Not an applicant.

## Methods for additional analyses (e.g., subgroup analyses) {20b}

Not an applicant.

### Methods in analysis to handle protocol non-adherence and any statistical methods to handle missing data {20c}

Sensitivity analysis was used for the lost causes, and the results of the last observation carried forward or the baseline observation carried forward were used for the sensitivity analysis of the measurement data. The sensitivity analysis of the counting data includes the analysis of the cases in the experimental group and the control group as the ineffective treatment and the best/worst case. In the worst-case analysis, the control group was treated successfully and the experimental group was treated as a failure.

### Plans to give access to the full protocol, participant level-data, and statistical code {31c}

Not an applicant.

### Oversight and monitoring

#### Composition of the coordinating center and trial steering committee {5d}

Not an applicant. The whole trial process will be monitored by the China Academy of Chinese Medical Sciences.

#### Composition of the data monitoring committee, its role, and reporting structure {21a}

Not an applicant. The data will be monitored by an independent statistician (the Institute of Clinical Basic Medicine, China Academy of Chinese Medical Sciences, China), who is not involved in the implementation of statistical analysis of the trial.

#### Adverse event reporting and harms {22}

Adverse events (AEs) are defined as any undesirable experience participants endure during the trial period, regardless of whether or not it is associated with the intervention. The participants will be instructed to report AEs to the research team, and a research nurse will monitor participants for potential occurrences of AEs. All details of AEs, such as time of occurrence, severity, management, and causality to the intervention, will be recorded in the case report forms. All AEs will be followed up from the beginning until resolution.

Current evidence has shown that AHP has a lower rate of adverse drug reactions (ADRs) [19]. However, our trial still pays more attention to possible ADRs, particularly associated with skin lesions (urticaria and pruritus) or other symptoms during the study period.

### Frequency and plans for auditing trial conduct {23}

The management department of the research institute or members of the ethics committee will review the progress of the trial every 3 months. The process will be independent of investigators and the sponsor.

### Plans for communicating important protocol amendments to relevant parties (e.g., trial participants, ethical committees) {25}

Any protocol modification changes such as eligibility criteria, outcomes, or analyses will be notified in writing to the management department of the research institute or members of the ethics committee.

### Dissemination plans {31a}

Research Report including results data will be promoted throughout the country through academic exchanges.

## Discussion

PONV is a common and unavoidable side effect of current forms of anesthesia. It has more impact on postoperative recovery and affects various aspects of life, including physiological, social, and psychological well-being. The Anesthesia Branch of the Chinese Medical Association provides an expert consensus including pharmacological and non-pharmacologic interventions [4]. The guidance document showed TCM therapies such as acupuncture and ginger which might relieve the symptoms of PONV still be under consideration. So the use of AHP for PONV after PLIF with general anesthesia needs rigorous scientific evidence.

This study has the potential to contribute to the development of an effective intervention to relieve PONV. It will also establish feasibility and provide preliminary evidence on the efficacy and safety of AHP in PONV. Based on the results of the study, future clinical practice should fully consider alternative and complementary therapy and include long-term administration and follow-up.

### Trial status

The version 2.0 protocol was formulated on 14 March 2018. The recruitment began on 18 May 2018. The trial is expected to complete in mid-2020.

## Abbreviations

PONV: Postoperative nausea and vomiting
PLIF: Posterior lumbar intervertebral fusion
RCT: Randomized controlled trial
AHP: Acupoint herbal plaster
APP: Acupoint placebo plaster
TCM: Traditional Chinese medicine
VAS: Visual analog scale
QOL: Quality of life
AE: Adverse event
ADR: Adverse drug reaction
